# High-Intensity Exercise Mitigates Cardiovascular Deconditioning During Long-Duration Bed Rest

**DOI:** 10.3389/fphys.2018.01553

**Published:** 2018-11-19

**Authors:** Martina A. Maggioni, Paolo Castiglioni, Giampiero Merati, Katharina Brauns, Hanns-Christian Gunga, Stefan Mendt, Oliver S. Opatz, Lea C. Rundfeldt, Mathias Steinach, Anika Werner, Alexander C. Stahn

**Affiliations:** ^1^Charité—Universitätsmedizin Berlin, Institute of Physiology, Center for Space Medicine and Extreme Environments Berlin, Berlin, Germany; ^2^Department of Biomedical Sciences for Health, Università degli Studi di Milano, Milan, Italy; ^3^IRCCS Fondazione Don Carlo Gnocchi, Milan, Italy; ^4^Université de Normandie, INSERM U 1075 COMETE, Caen, France; ^5^Department of Psychiatry, Perelman School of Medicine, University of Pennsylvania, Philadelphia, PA, United States

**Keywords:** heart rate variability, hemodynamics, countermeasure, cardiovascular deconditioning, posture, long-term bed rest

## Abstract

Head-down-tilt bed rest (HDT) mimics the changes in hemodynamics and autonomic cardiovascular control induced by weightlessness. However, the time course and reciprocal interplay of these adaptations, and the effective exercise protocol as a countermeasure need further clarification. The overarching aim of this work (as part of a European Space Agency sponsored long-term bed rest study) was therefore to evaluate the time course of cardiovascular hemodynamics and autonomic control during prolonged HDT and to assess whether high-intensity, short-duration exercise could mitigate these effects. A total of *n* = 23 healthy, young, male participants were randomly allocated to two groups: training (TRAIN, *n* = 12) and non-training (CTRL, *n* = 11) before undergoing a 60-day HDT. The TRAIN group underwent a resistance training protocol using reactive jumps (5–6 times per week), whereas the CTRL group did not perform countermeasures. Finger blood pressure (BP), heart rate (HR), and stroke volume were collected beat-by-beat for 10 min in both sitting and supine positions 7 days before HDT (BDC−7) and 10 days after HDT (R+10), as well as on the 2nd (HDT2), 28th (HDT28), and 56th (HDT56) day of HDT. We investigated (1) the isolated effects of long-term HDT by comparing all the supine positions (including BDC−7 and R+10 at 0 degrees), and (2) the reactivity of the autonomic response before and after long-term HDT using a specific postural stimulus (i.e., supine vs. sitting). Two-factorial linear mixed models were used to assess the time course of HDT and the effect of the countermeasure. Starting from HDT28 onwards, HR increased (*p* < 0.02) and parasympathetic tone decreased exclusively in the CTRL group (*p* < 0.0001). Moreover, after 60-day HDT, CTRL participants showed significant impairments in increasing cardiac sympathovagal balance and controlling BP levels during postural shift (supine to sitting), whereas TRAIN participants did not. Results show that a 10-day recovery did not compensate for the cardiovascular and autonomic deconditioning following 60-day HDT. This has to be considered when designing rehabilitation programs—not only for astronauts but also in general public healthcare. High-intensity, short-duration exercise training effectively minimized these impairments and should therefore deserve consideration as a cardiovascular deconditioning countermeasure for spaceflight.

## Introduction

Upcoming deep space missions such as Martian expeditions will require exposure to up to 1,000 days in microgravity (Horneck, 2006). Space agencies are thus investigating adverse health effects of long-term missions and their possible countermeasures in order to reduce detrimental consequences for astronaut health (Aubert et al., 2016; Bergouignan et al., 2016; Frippiat et al., 2016; White et al., 2016; Lang et al., 2017). Space analogs simulating prolonged gravity changes therefore play a crucial role (Ploutz-Snyder, 2016). Bed rest with −6 degrees head-down tilt (HDT) is one of the best conditions to mimic the effect of long-term weightlessness on the human body—even within the limitations of this model. HDT shifts fluids from the lower domain to the upper region of the body, similar to the fluid centralization observed in spaceflight (Pavy-Le Traon et al., 2007; Hargens and Vico, 2016; Watenpaugh, 2016). Bed rest models also allow for the investigation of some of the effects of immobilization, secondary to hospitalization, and physical inactivity. Indeed, elderly patients spend over 80% of their hospital admission confined to their bed (Vernikos and Schneider, 2010; Baczynska et al., 2016), and physical inactivity is one of the leading causes of death in Western countries (Blair, 2009). Therefore, investigating the physiological consequences of physical inactivity and designing effective countermeasures is essential for planning future long-term space missions as well as for public health and rehabilitation purposes. Weightlessness negatively affects several physiological functions. For example, it can cause deconditioning of the cardiovascular system, which may be characterized by higher resting heart rate with altered autonomic control associated with orthostatic intolerance (Blomqvist et al., 1994; Sigaudo et al., 1998; Fortrat et al., 2001; Custaud et al., 2002). Despite research spanning at least four decades on weightlessness-associated cardiovascular alterations (Pavy-Le Traon et al., 2007; Hargens and Vico, 2016), the exact time courses of changes in hemodynamic regulation and autonomic cardiovascular control induced by long-term spaceflight are not fully understood (Liu et al., 2015; Aubert et al., 2016). Moreover, several countermeasures for cardiovascular deconditioning have already been tested (e.g., volume loading, lower-body negative pressure, hypergravity; Wang et al., 2011; Stenger et al., 2012; Jeong et al., 2014; Li et al., 2017), but exercise is the most investigated countermeasure (Blaber et al., 2009; Petersen et al., 2016; Ploutz-Snyder, 2016). However, despite the consensus on physical activity as a countermeasure, the type and intensity of the exercise are undergoing further investigation. Common exercise countermeasures include aerobic (Pagani et al., 2009; Cavanagh et al., 2017; Demontis et al., 2017) and resistive exercise (Holt et al., 2016; Demontis et al., 2017), as well as in combination with whole-body vibration (Belavý et al., 2010). Recent findings show high-intensity interval training (HIIT) to be salutary in cardiovascularly compromised persons (Ramos et al., 2015; Fleg, 2016; Hussain et al., 2016), improving aerobic capacity, endothelial, and left-ventricular function, vasomotor function, and blood pressure (Hussain et al., 2016). So far, however, HIIT has rarely been implemented to counteract cardiovascular deconditioning and orthostatic intolerance in microgravity settings (Hughson et al., 1994; Greenleaf, 1997; Hastings et al., 2012; Hargens et al., 2013). This study therefore aimed to evaluate whether short-duration HIIT is an effective countermeasure against cardiovascular deconditioning and orthostatic intolerance induced by 60 days of head-down-tilt bed rest. To achieve this aim, we compared subjects doing HIIT with a control group and investigated 1) the time course of hemodynamic changes and adaptations of the cardiovascular autonomic control during 60-day HDT, and 2) the cardiovascular response to a postural test performed before and after the bed rest confinement. As for the HIIT exercise, we administered specific lower body resistance training that provides neuromuscular force solicitation: the reactive jump protocol in a sledge jump system (Kramer et al., 2010, 2017b).

## Methods

This research was performed as part of the European Space Agency (ESA) sponsored study “**R**eactive jumps in a **S**ledge jump system as countermeasure during **L**ong-term bed rest—RSL Study” at the DLR :*envihab* (German Aerospace Agency (DLR), Cologne, Germany), between 2015 and 2016. Details related to the core project design, recruitment, randomization of volunteers, and training protocol are reported elsewhere (Kramer et al., 2017a,b). The study was conducted in accordance with the Declaration of Helsinki for Medical Research Involving Human Subjects (revision October 2013) and was approved by the ethics committee of the Northern Rhine Medical Association in Düsseldorf, Germany (see Kramer et al., 2017a). After the purpose, procedures, and known risks of the tests had been explained to the participants, each participant gave written informed consent. In brief, the study consisted of 15 days of baseline data collection (BDC−15 through BDC−1), 60 days of HDT bed rest (HDT1 through HDT60), and 15 days of recovery (R+0 through R+14). During the 60 days of −6 degrees HDT, the reactive jump training was administered as a countermeasure in one randomly selected subsample (TRAIN: training), whereas the other subsample (CTRL: control) did not perform any physical training (see section subjects below). The training protocol was performed using a sledge jump system (Novotec Medical GmbH, Pforzheim, Germany) composed of a lightweight sledge sliding on rails. Cylinders pull the sledge toward the plates with force exerted on the subject by adjusting the pressure settings within the cylinders. The participant was fixed supine to the sledge with shoulder straps and with feet on force plates. Participants would then perform countermovement jumps while receiving feedback on jump height and peak force from a monitor. Participants in the TRAIN group underwent the training protocol starting from HDT1. Each training session consisted of repetitive jumps and different series of countermovement jumps with an average load equal to or above 80% of the participant's body weight. Sessions lasted about 20 min, including preparation. Training took place in the afternoon between 2 and 6 pm, 5–6 times per week, for a total of 48 sessions during the 60-day bed rest. A comprehensive description of the sledge system, the training method, and timeline is reported elsewhere (Kramer et al., 2017a,b).

### Subjects

Data were collected from 23 young, healthy, male participants (baseline: age 29 ± 6 [m ± SD] years, weight 77 ± 7 kg, height 181 ± 6 cm), who were not involved in competitive or professional sport activities at the time of the study (see Kramer et al., 2017a,b for details on inclusion and exclusion criteria). Participants were randomly allocated to a training group (TRAIN, *n* = 12, age 30 ± 7 years, weight 78 ± 7 kg, height 181 ± 7 cm) or to a control group (CTRL, *n* = 11, age 28 ± 6 years, weight 76 ± 8 kg, height 181 ± 5 cm), and were matched based on anthropometry (Kramer et al., 2017a). One subject of the TRAIN group and one of the CTRL group were re-ambulated after 49 and 50 days of HDT (instead of 60 days), respectively, for medical reasons (Kramer et al., 2017a). Notably, this did not affect their completion of the recovery phase and all the scheduled measurements were collected. Accordingly, these subjects were included in the data analysis.

### Data collection

**Figure 1:**
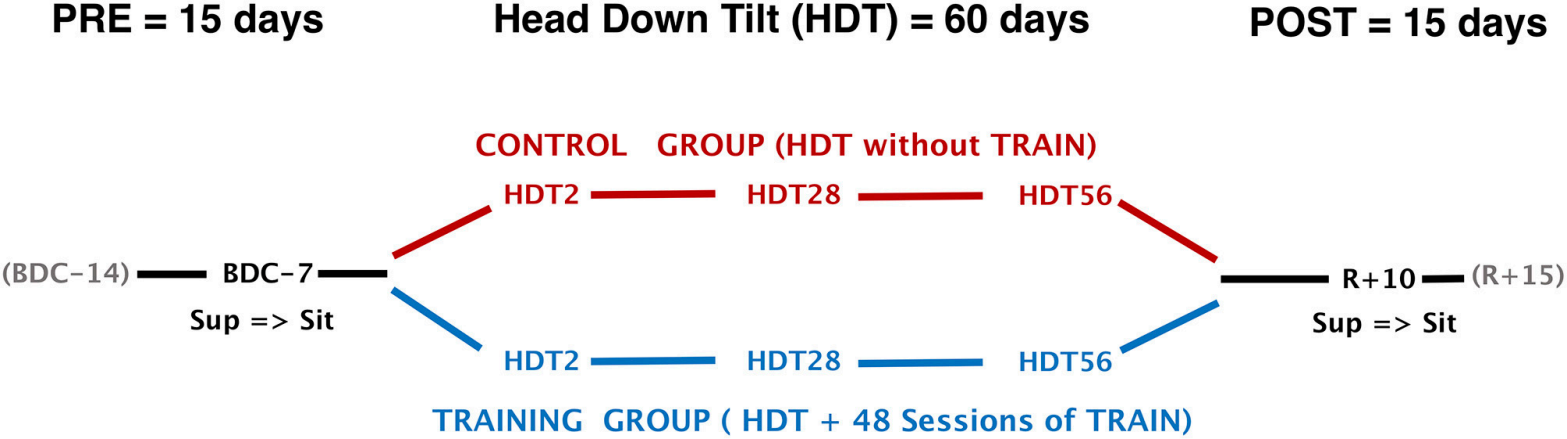
Study design and timeline of data collection. HDT, head-down tilt; TRAIN, reactive jumps training; BDC−7, Baseline Data Collection (7 days before start of HDT); R+10, Recovery data collection (10 days after the end of HDT); Sup, measurements in supine position; Sit, measurements in sitting position.

To evaluate short-, mid-, and long-term exposure to HDT, autonomic cardiovascular and hemodynamic data were collected on the 2nd (HDT2), on the 28th (HDT28), and on the 56th (HDT56) day of HDT. To evaluate the response to a postural stimulus, we also collected the identical data 7 days before the start of HDT (BDC−7) and 10 days after the end of HDT (R+10, R+0 being the first day of recovery) in both sitting and supine positions (Figure 1). In each session, a time series of physiological data were recorded for 10 min. During the lay-to-sit challenge (in BDC−7 and R+10) data were first recorded for 10 min in the supine position and then for an additional 10 min immediately after the change of posture to the sitting position. Recordings included blood pressure at the finger artery (BP) measured via continuous finger plethysmography and one-lead electrocardiogram (ECG). Both were sampled at 200 Hz. Beat-by-beat stroke volume (SV), cardiac output (CO), and total peripheral resistance (TPR) were obtained using impedance cardiography (TensoScreen®, Medis Germany). All measurement sessions were conducted between 9 and 12 am (before lunch), at least 18 h after the previous training session. Participants were required to avoid caffeine consumption during the 4 h leading up to the measurements, and were instructed not to move, talk, or fall asleep during the recordings.

### Data analysis

An expert operator visually inspected the ECG and BP signals, identifying and manually removing possible artifacts and premature beats. Beat-by-beat time series of normal-to-normal R-R intervals were derived from the ECG tracing for heart rate variability (HRV) analysis. Beat-by-beat values of systolic BP (SBP) and diastolic BP (DBP) were obtained from the BP signal. Beat-to-beat series of R-R intervals and DBP values were interpolated linearly at 10 Hz and resampled at 5 Hz. The Welch periodogram was estimated by splitting the resampled series in 90% overlapping Hann windows of 240 s in duration, computing the FFT spectrum in each window, and by averaging the spectra over all the windows. The final periodogram was smoothed with a broadband procedure that averages adjacent spectral lines with a moving average filter whose order increases with the frequency from 3 to 11 (Di Rienzo et al., 1996). Following the guidelines on HRV analysis (Task Force of the European Society of Cardiology the North American Society of Pacing Electrophysiology, 1996), the power spectrum of R-R intervals was integrated over a very-low-frequency (VLF, 0.005–0.04 Hz), a low-frequency (LF, 0.04–0.15 Hz), and a high-frequency band (HF, 0.15–0.40 Hz). The ratio between LF and HF powers was calculated as an index of cardiac sympathovagal balance (i.e., LF/HF). The LF power was also derived for the DBP spectrum as an index of sympathetic modulations of the vascular tone (Castiglioni et al., 2007). The short-term fractal index DFA1 was estimated by applying detrended fluctuation analysis to the beat-by-beat R-R interval series (Peng et al., 1995) and by considering block sizes not larger than 16 beats. DFA1 reflects changes in the cardiac autonomic tone, which increases when the sympathovagal balance increases or the vagal tone decreases (Tulppo et al., 2001; Castiglioni et al., 2011). The mean breathing rate was evaluated as the central frequency of the power spectrum of ECG-derived respiration (EDR) signal. The EDR signal reflects the respiratory movements of the thorax as modulations of the amplitude of the QRS complex (Schmidt et al., 2017).

### Statistics

Descriptive statistics have been reported as means and standard deviations (m ± SD) unless stated otherwise. To evaluate the time course of cardiovascular changes during and after bed rest compared to baseline, we expressed data of HDT2, HDT28, HDT56, and R+10 as percentage changes from BDC−7 by dividing the values recorded in each HDT time point by the corresponding value measured in BDC−7 supine (which therefore corresponds to the 100% reference). A log transformation was applied to frequency-domain indices to attain normal distribution (Castiglioni et al., 1999). Because of the properties of the logarithm, the normalized variables were expressed as the difference between the log-transformed value in each time point and the log-transformed value in the baseline, which therefore corresponds to the reference zero level. Two-factorial linear mixed models were then used to assess the time course of cardiovascular changes in supine position within and between subjects. Subjects were entered as random factors and bed rest Time (HDT2, HDT28, HDT56, and R+10) and intervention Group (CTRL and TRAIN) were included as fixed factors. Significant effects of Time and Group or their interaction were followed up using contrasts (with BDC−7 as a reference for Time). When only the factor Time was significant, contrasts were performed irrespective of the intervention (i.e., CTRL and TRAIN groups were pooled).

As for the postural test (i.e., the shift from supine to sitting) performed before and after HDT, variables were expressed as *delta scores*. The *delta score* corresponds to the difference (Δ) between the value measured before bed rest (at BDC−7) and the respective value measured after bed rest (at R+10) per group (CTRL and TRAIN) and position (supine and sitting). Therefore, *delta scores* are changes from baseline values (i.e., BDC−7) and express the effects of bed rest according to each group and each posture. Mixed models were used to assess within-participant and between-participant differences in *delta scores*. Body position and intervention group were included as fixed factors and subjects as random factors. When the factor Posture, or Group, or their interaction was significant, contrasts were performed to follow up on single comparisons and corrected for multiple comparisons by a sequentially rejective correction procedure. To test whether bed rest had a significant effect in a given group and posture, contrasts were used to determine whether each *delta score* differed significantly from zero (non-zero contrasts). Covariance matrices were determined by restricted maximum likelihood (REML) estimation. *P*-values were obtained using Satterthwaite's approximation for denominator degrees of freedom. Normality and homogeneity were checked via visual inspections of plots of residuals against fitted values. The level of significance was set at α = 0.05 (two-sided) for all testing. All comparisons were corrected for multiple comparisons using a sequentially rejective correction procedure (Hochberg, 1988). To maximize sensitivity for detecting true differences while maintaining control over family-wise Type I errors, we followed the recommendation of choosing smaller, more focused families rather than broad ones (Westfall et al., 2011). All statistical analyses and graphical illustrations were carried out using the software package R (R Core Team, 2016) Mixed models were run using the packages lme4 and lmerTest (Bates et al., 2014; Alexandra Kuznetsova et al., 2016). Adjusted means were calculated using the lsmeans package (Lenth, 2016) and htmlTable (Max Gordon, 2017). Figures were created using ggplot2, ggpubr, and cowplot (Wickham, 2016; Alboukadel Kassambara, 2017; Claus O. Wilke, 2017).

## Results

### Time course of hemodynamic variables during HDT

**Figure 2:**
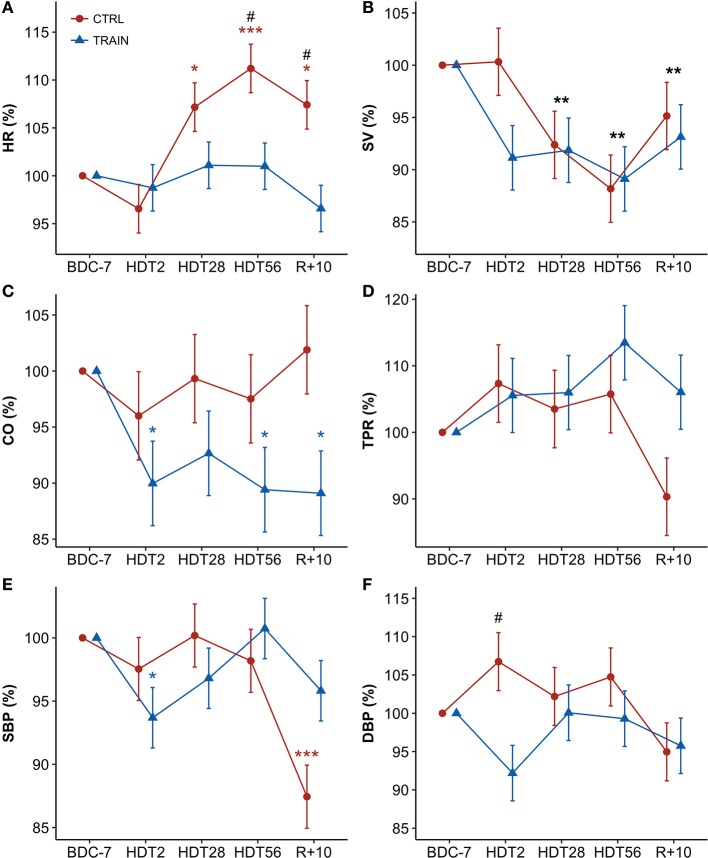
**(A–F)** Time courses of hemodynamic variables for CTRL (red circles) and TRAIN (blue triangles) groups. Measurements time points in supine position: 7 days before (BDC−7) and 10 days after (R+10) HDT, and on the 2nd (HDT2), 28th (HDT28) and 56th (HDT56) day of HDT. Data are presented as marginal means ± SE of percent changes from baseline values (with BDC−7 supine = 100%) at each time point. Colored asterisks indicate significance compared to BDC−7 within the single group. Black asterisks show significant difference from baseline in the whole sample of participants (when only the factor Time - and not the factor Group or their interaction - is significant). The pound sign denotes significant differences between groups at each time point. **(A)** HR, Heart Rate; **(B)** SV, Stroke Volume; **(C)** CO, Cardiac Output; **(D)** TPR, Total Peripheral Resistance; **(E)** SBP, Systolic Blood Pressure; **(F)** DBP, Diastolic Blood Pressure. ^***^p < 0.001, ^**^p < 0.01, ^*^p < 0.05, ^#^*p* < 0.05.

Figure 2 shows percent changes in hemodynamic values from baseline (BDC−7 supine) measured during HDT and recovery. Table 1 reports the results of the linear mixed models analysis. The factor Time and its interaction with Group were significant for HR. Accordingly, HR was higher than at baseline from HDT28 up to the recovery phase (R+10) in CTRL participants, while not exhibiting changes in the TRAIN group. In particular, the difference between groups in HR changes was statistically significant near the end of bed rest (HDT56) and during recovery (R+10). Time was also a significant factor for SV, which decreased during HDT, recovering only partially in R+10. The factor Group was marginally significant for CO, with values lower than baseline for only the TRAIN group. The factor Time and its interaction with Group were highly significant for SBP. Figure 2 shows a marked fall in SBP during recovery in the CTRL group only. DBP showed a marginal significance for the interaction between Group and Time. No factors were significant for TPR (Figure 2) and EDR; the respiratory rate did not change significantly during and after HDT.

**Table 1:** HDT time course: significance of Time and Group factors and their interaction based on linear mixed models analysis (see text for abbreviations).

	**Significance p**		
	**Time**	**Group**	**Time × Group**
**HR**	< 0.001	< 0.05	< 0.01
**SV**	< 0.05	0.43	0.16
**CO**	0.67	0.055	0.65
**SBP**	< 0.001	0.72	< 0.01
**DBP**	0.12	0.18	0.06
**TPR**	0.09	0.31	0.26
**RRI SPECTRAL POWERS:**			
**log HF**	< 0.001	0.19	< 0.01
**log LF**	< 0.001	0.74	< 0.01
**log VLF**	< 0.001	0.75	0.20
**log LF/HF**	0.055	0.28	0.61
**DFA1**	< 0.05	0.15	0.06
**DBP SPECTRAL POWERS:**			
**log LF**	0.38	0.37	0.21

### Time course of autonomic indices during HDT

**Figure 3:**
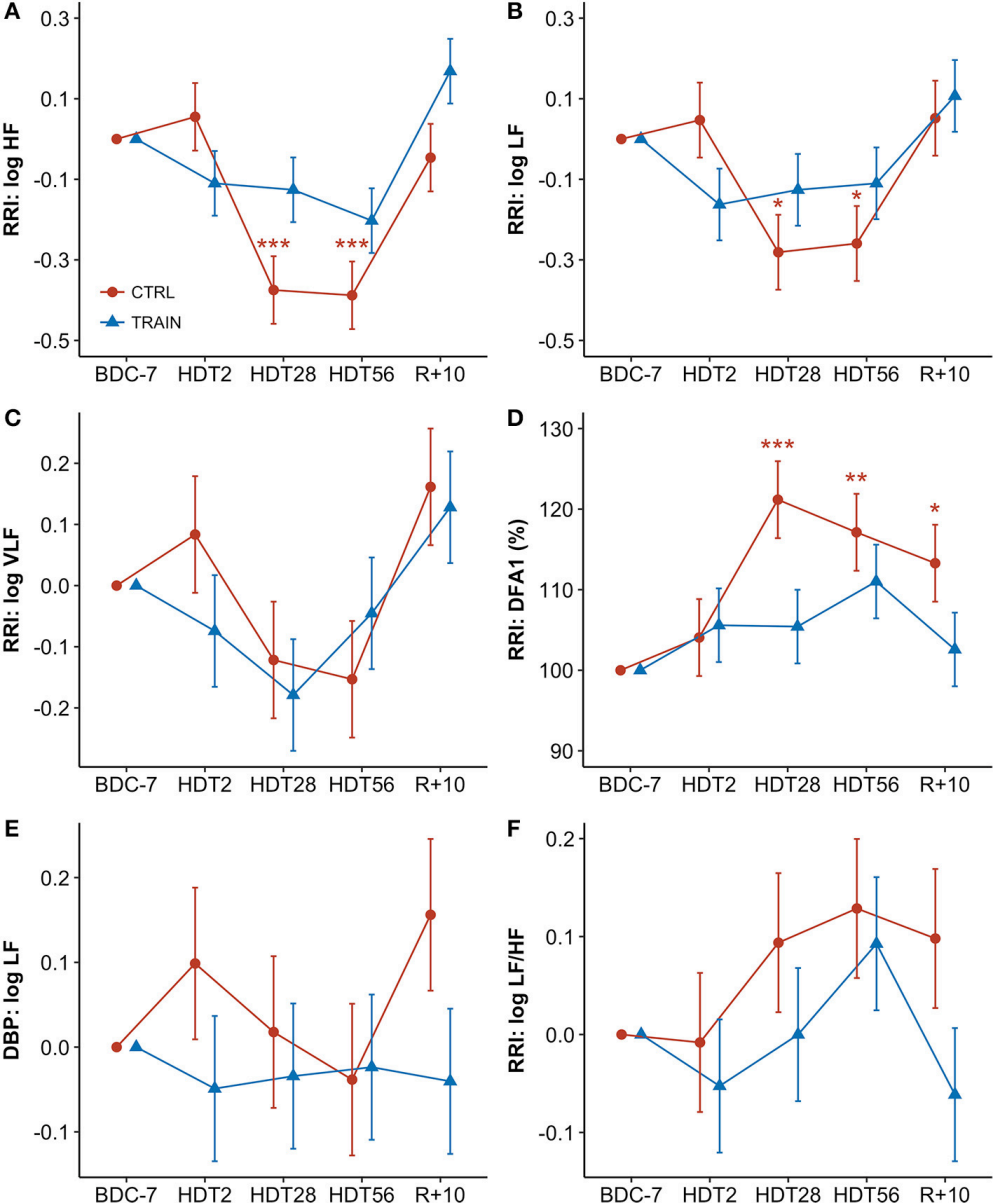
**(A–F)** Time courses of autonomic variables for CTRL (red circles) and TRAIN (blue triangles) groups. Measurements time points in supine position: 7 days before (BDC−7) and 10 days after (R+10) HDT, and on the 2nd (HDT2), 28th (HDT28) and 56th (HDT56) day of HDT. Data are presented for each time point as marginal mean ± SE of differences with respect to baseline supine position (for spectral powers, BDC−7 supine = 0) or of percent changes from baseline supine position (for DFA1, BDC−7 supine = 100%). Colored asterisks indicate significance compared to BDC−7 within the single group. RRI, R-R intervals; **(A)** HF, High-Frequency power; **(B)** LF, Low-Frequency power; **(C)** VLF, Very-Low-Frequency power; **(D)** DFA1, short-term fractal index by detrended fluctuations analysis; **(E)** LF of DBP: Low-Frequency power of Diastolic Blood Pressure; **(F)**: LF/HF, Low-Frequency to High-Frequency powers ratio ^***^p < 0.001, ^**^p < 0.01, ^*^p < 0.05.

Figure 3 shows percent changes in autonomic indices from baseline. Table 1 shows the results of the linear mixed models. Since percent changes of spectral powers were log-transformed before the statistical test, they are reported as differences vs. zero, i.e., the baseline reference (see Methods). The factor Time and the interaction between Time and Group were highly significant for the HF and LF powers of RRI (Table 1). Both these powers had values lower than baseline at HDT28 and HDT56 for the CTRL group only. Time was a significant factor and the interaction between Time and Group was close to the 5% significance threshold. This was also the case for DFA1, whose profile mirrored the profile of the HF power, with a significant increase at HDT28 and HDT56 for the CTRL group only. In this case, however, the increase was consistently statistically significant during recovery as well. As for the LF/HF powers ratio, Time was the only factor close to statistical significance (Table 1). No factors were significant for the LF power of DBP.

### Hemodynamic response to the postural test

**Figure 4:**
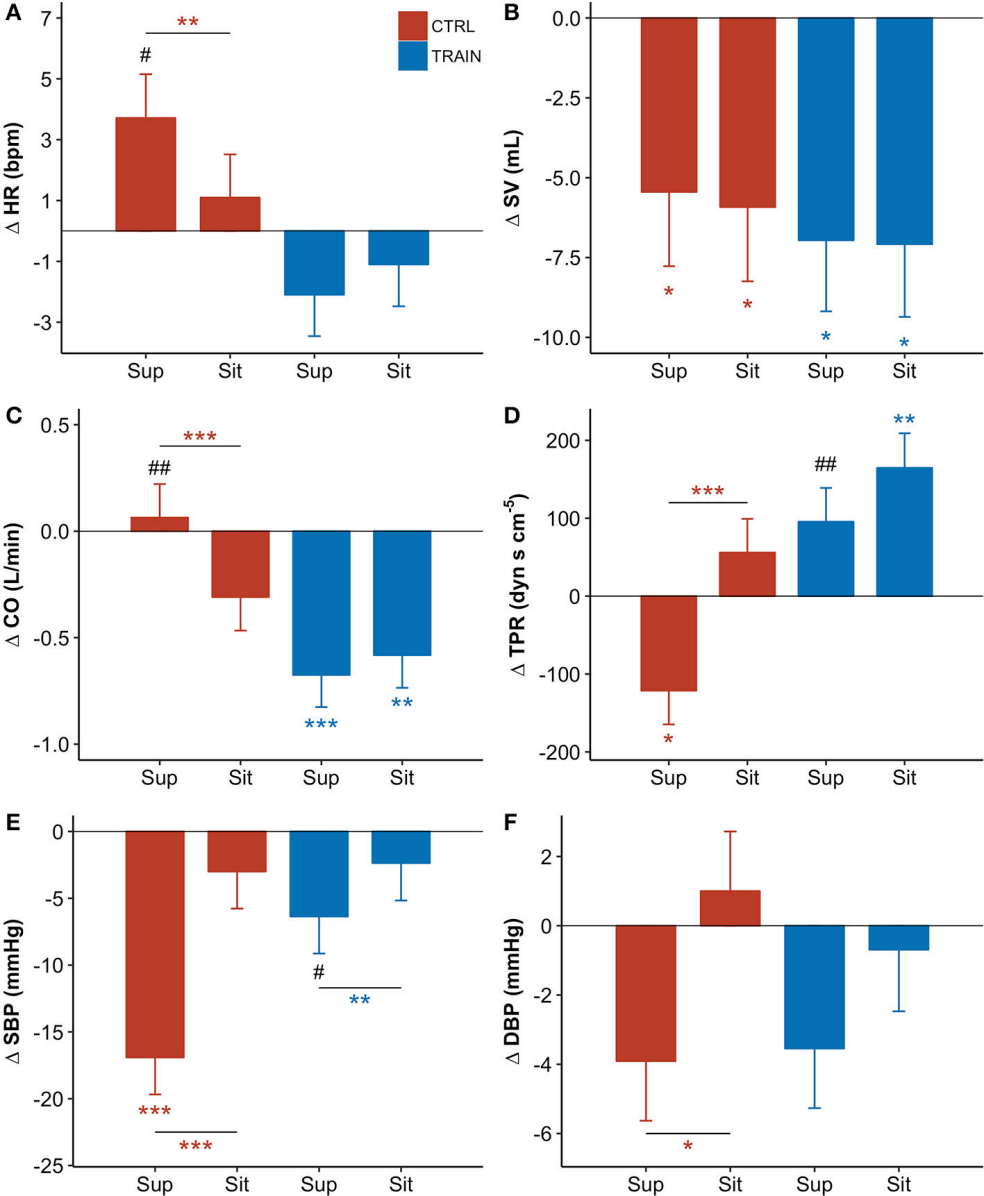
**(A–F)** Delta scores of hemodynamic variables, i.e., the difference (Δ) between the value measured before bed rest (at BDC−7) and the corresponding value measured after bed rest (at R+10, see text) for the supine (Sup) and the sitting (Sit) position: adjusted means ± SE. For each panel: control group (CTRL) red bars on the left-hand side, and training group (TRAIN) blue bars on the right-hand side. Asterisks above whiskers indicate *delta scores* significantly different from zero. Horizontal lines with asterisks indicate significant differences between supine and sitting *delta scores* within the same group. Pound signs indicate significant differences between groups at the same position. **(A)** HR, Heart Rate; **(B)** SV, Stroke Volume; **(C)** CO, Cardiac Output; **(D)** TPR, Total Peripheral Resistance; **(E)** SBP, Systolic Blood Pressure; **(F)** DBP, Diastolic Blood Pressure. ^***^p < 0.001, ^**^p < 0.01, ^*^p < 0.05, *^##^p* < 0.01, ^#^*p* < 0.05.

Table 2 reports descriptive statistics of hemodynamic data in supine and sitting positions at BDC−7 and R+10. Not only before but also after bed rest, the shift from supine to sitting posture appears associated with an increase in HR, DBP, and TPR, and with a decrease in SV and CO in both groups. However, the bed rest had a different effect on the amplitude of the changes in the two groups, as reported in Figure 4 (i.e., *delta scores* of hemodynamic variables) and Table 3 (i.e., factors significance based on linear mixed models). The HR *delta score* of the CTRL group in supine position was positive and significantly higher than in sitting position (Figure 4A), suggesting that bed rest increased HR more in the supine than the sitting position in the CTRL group. The factor Group and its interaction with Posture were significant (Table 3), and bed rest did not increase HR in the TRAIN group (*delta scores* were negative). A significantly different *delta score* between groups in the supine position was also found. Bed rest decreased SV in both groups (Figure 4B), independent of posture (Table 3). CO (the product of HR and SV) reflected the combination of HR and SV *delta scores*. Both factors and their interaction were significant (Table 3) and *delta scores* differed between the groups in the supine position and between positions in the CTRL group (Figure 4C).

**Table 2:** Postural test: mean (SD) in the supine (Sup) and sitting (Sit) position before (BDC−7) and after (R+10) bed rest (see text for abbreviations).

		***CTRL***		***TRAIN***	
	**Time**	**Sup**	**Sit**	**Sup**	**Sit**
**HR (bpm)**	BDC−7	58.5(7.4)	69.1(8.3)	64.2(9.1)	70.4(7.8)
	R+10	62.7(7.6)	70.2(5.9)	62.1(10.5)	68.2(7.8)
**SV (mL)**	BDC−7	106.7(18.7)	81.5(14.0)	101.6(14.1)	76.8(12.5)
	R+10	101.3(17.9)	75.6(11.8)	94.6(15.9)	70.9(9.0)
**CO (L/min)**	BDC−7	6.1(1.1)	5.5(1.0)	6.5(1.1)	5.3(0.8)
	R+10	6.2(1.2)	5.2(0.8)	5.8(1.3)	4.7(0.6)
**SBP (mmHg)**	BDC−7	129.5(10.0)	125.4(10.0)	129.7(8.6)	127.6(9.6)
	R+10	112.5(7.8)	122.4(7.0)	124.2(9.3)	126.6(8.1)
**DBP (mmHg)**	BDC−7	70.5(7.5)	76.6(7.1)	76.0(8.6)	81.6(9.6)
	R+10	66.5(4.8)	77.6(7.5)	72.7(10.0)	81.0(5.4)
**TPR (dyn s cm**^5^**)**	BDC−7	1148(235)	1318(249)	1154(246)	1395(209)
	R+10	1026(226)	1379(182)	1223(299)	1559(188)
**EDR (Hz)**	BDC−7	0.27(0.03)	0.29(0.03)	0.27(0.04)	0.27(0.03)
	R+10	0.27(0.02)	0.27(0.03)	0.27(0.03)	0.27(0.02)
**RRI:**					
**log HF (log ms**^2^**)**	BDC−7	2.85(0.50)	2.38(0.50)	2.58(0.38)	2.40(0.25)
	R+10	2.81(0.49)	2.69(0.45)	2.75(0.47)	2.54(0.34)
**log LF (log ms**^2^**)**	BDC−7	3.10(0.40)	3.02(0.31)	3.03(0.28)	3.09(0.34)
	R+10	3.15(0.29)	3.08(0.28)	3.14(0.21)	3.17(0.25)
**log VLF (log ms**^2^**)**	BDC−7	3.18(0.39)	3.22(0.37)	3.09(0.35)	2.97(0.21)
	R+10	3.34(0.40)	3.30(0.45)	3.22(0.40)	3.15(0.31)
**log LF/HF**	BDC−7	0.46(0.07)	0.65(0.48)	0.39(0.05)	0.70(0.29)
	R+10	0.34(0.36)	0.39(0.45)	0.38(0.32)	0.63(0.30)
**DFA1**	BDC−7	0.81(0.15)	1.09(0.20)	0.87(0.16)	1.04(0.11)
	R+10	0.90(0.12)	0.96(0.16)	0.89(0.15)	1.03(0.11)
**DBP:**					
**log LF (log mmHg**^2^**)**	BDC−7	1.28(0.25)	1.20(0.33)	1.32(0.23)	1.21(0.14)
	R+10	1.43(0.19)	1.26(0.29)	1.28(0.19)	1.26(0.15)

**Table 3:** Postural test: significance of Group and Posture factors and their interaction based on linear mixed models analysis (see text for abbreviations).

	**Significance p**		
	**Group**	**Posture**	**Group x Posture**
**HR**	< 0.05	0.16	< 0.01
**SV**	0.66	0.82	0.89
**CO**	< 0.05	0.053	< 0.01
**SBP**	0.16	< 0.001	< 0.001
**DBP**	0.76	< 0.01	0.38
**TPR**	< 0.05	< 0.001	< 0.05
**RRI SPECTRAL POWERS:**			
**log HF**	0.96	< 0.01	< 0.001
**log LF**	0.55	0.83	0.48
**log VLF**	0.99	0.26	0.23
**log LF/HF**	0.12	0.07	0.46
**DFA1**	0.48	< 0.001	< 0.001
**DBP SPECTRAL POWERS:**			
**log LF**	0.13	0.09	< 0.001

The factors Posture, Group, and their interaction were also significant for TPR (Table 3). The negative TPR *delta score* of CTRL participants in the supine position indicates that bed rest decreased supine TPR only in the CTRL group (Figure 4D). Posture and its interaction with Group were also significant factors for SBP (Table 3). Bed rest decreased SBP more in supine than in sitting position and markedly more in the CTRL group (Figure 4E). Effects of bed rest were less pronounced on DBP (Figure 4F) than on SBP.

### Autonomic response to the postural test

**Figure 5:**
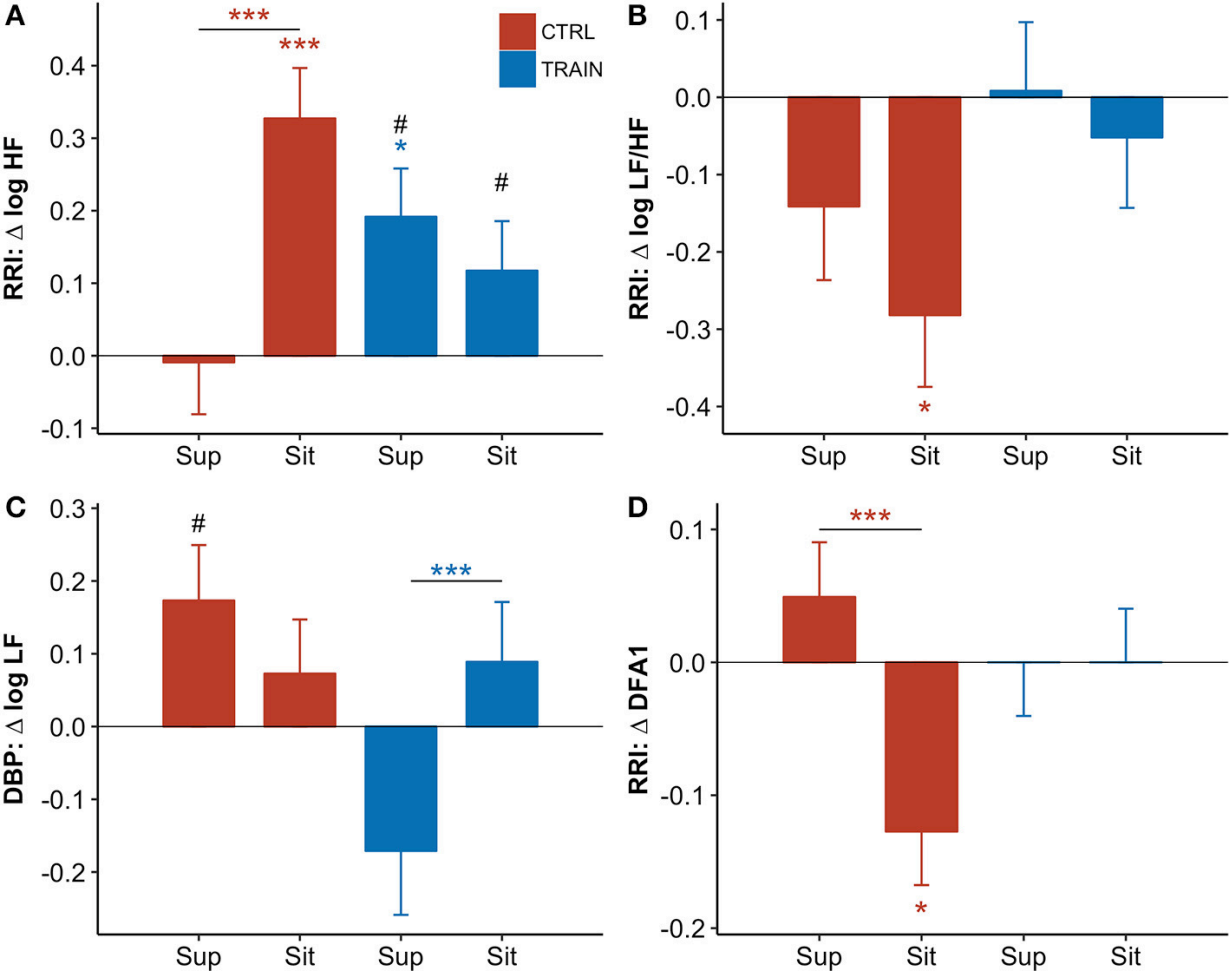
**(A–D)** Delta scores of autonomic variables, i.e., the difference (Δ) between the value measured before bed rest (at BDC−7) and the corresponding value measured after bed rest (at R+10, see text) for the supine (Sup) and the sitting (Sit) position: adjusted means ± SE. For each panel: control group (CTRL) red bars on the left-hand side, and training group (TRAIN) blue bars on the right-hand side. Asterisks above whiskers indicate *delta scores* significantly different from zero. Horizontal lines with asterisks indicate significant differences between supine and sitting *delta scores* within the same group. Pound signs indicate significant differences between groups at the same position. RRI, R-R intervals; **(A)** HF, High-Frequency power; **(B)** LF/HF, Low-Frequency to High-Frequency powers ratio; **(C)** LF, Low-Frequency power; **(D)** DFA1, short-term fractal index by detrended fluctuations analysis. ^***^p < 0.001, ^*^p < 0.05, ^#^*p* < 0.05.

The shift from supine to sitting position was associated with an increase in the LF/HF powers ratio and DFA1, and a decrease in the HF power of RRI and the LF power of DBP—both before and after bed rest (Table 2). However, the prolonged bed rest period influenced these changes differently in the two groups (Figure 5). In fact, after bed rest, the HF power of RRI in supine position only increased in the TRAIN group (Figure 5A), and the DFA1 in sitting position only decreased in the CTRL group (with differences in *delta scores* between positions found only in the CTRL group, Figure 5D). Furthermore, bed rest decreased the sitting LF/HF index in CTRL participants only and influenced the supine LF power of DBP differently between groups (i.e., the *delta score* was positive for CTRL participants and negative for TRAIN participants, Figures 5B and 5C).

## Discussion

Our study investigated the alterations induced by 60 days of HDT in hemodynamics and autonomic modulations of heart rate. To the best of our knowledge, this study involved the highest number of participants to investigate these specific effects during long-term HDT bed rest so far. Our main findings show that: (1) HR progressively increased some days after the start of the HDT; (2) changes in SV and HR vagal modulations appeared almost synchronously; and (3) alterations in these variables and SBP were detectable several days after the end of bed rest, indicating persistent cardiovascular deconditioning. Furthermore, this high-intensity/short-duration exercise alleviated the cardiovascular deconditioning, counteracting the autonomic alterations and improving recovery. Although the adopted exercise protocol mainly involved the lower part of the body, it likely influenced central mechanisms of cardiac modulation and appears to be a promising countermeasure for long-term spaceflight missions. Different exercise-based countermeasures have been tested so far to reduce cardiovascular deconditioning. A previous study showed that low-magnitude whole body vibration with resistive exercise prevented the increase of the autonomic index of cardiac sympathovagal balance after an HDT period lasting 60 days (as was the case in our study) without, however, improving orthostatic tolerance (Coupé et al., 2011). Other studies were different from the present in their design and duration. Nonetheless, these studies highlight the crucial role of exercise as a countermeasure against cardiovascular deconditioning. The intermittent exposure to hypergravity coupled with ergometric exercise limited the decrease in parasympathetic activity after 14 days of HDT (Iwasaki et al., 2005). Daily rowing ergometry and biweekly strength exercise training after 5 weeks of HDT prevented orthostatic intolerance only when combined with volume loading (Hastings et al., 2012). Finally, supine cycling could counteract orthostatic intolerance after 18-day bed rest only in combination with plasma volume restoration (Shibata et al., 2010). However, the vast range of different study designs and countermeasures complicate a direct comparison with the sledge jump training protocol.

### Time course of hemodynamic variables and autonomic indices during HDT

We described the time course of long-term HDT adaptations by comparing values during HDT with baseline measures in the supine position (0 degrees). Compared to sitting, the recumbent position is characterized by a fluid shift to the upper body, which increases stroke volume and stimulates baroreceptors and volume receptors, inducing cardiac vagal activation. By choosing this reference we could describe the isolated effect of the prolonged −6 degrees bed rest and this makes our results comparable with the cardiovascular changes observed during mid- and long-term spaceflight (Baevsky et al., 2007; Di Rienzo et al., 2008; Demontis et al., 2017). In the CTRL group, HR significantly increased from baseline starting from the 28th day of HDT and remained higher than baseline on the 56th day, in the recovery phase, and 10 days after the end of bed rest (R+10 supine; Figure 2A). A similar pattern was observed in another 60-day HDT study (Liu et al., 2015). Interestingly, this pattern was missing in the TRAIN group, where HR remained stable during and after HDT. The HR increase is a crucial feature of cardiovascular deconditioning, and the lack of this feature in the TRAIN group is a clear marker of the efficacy of the proposed training protocol as a cardiovascular countermeasure. A question arises about the type of training we adopted as short duration HIIT is not usually considered to act as a *vagal enhancer*. However, some data confirm that also this type of training may induce a parasympathetic adaptation of HR when performed in the supine position (Kiviniemi et al., 2014). An alternative hypothesis for the unchanged HR in TRAIN group after 28 days of bed rest might be constant increments in left atrial volume that could have induced a bradycardic response by stretching the sinus node. However, this unlikely occurred in our study because we found a significant reduction in SV in both groups (Figure 2A). SV depends on contractility, on arterial blood pressure, and on atrial pressure. Since contractility did not change (as sympathetic indices of HRV remained stable in the TRAIN group) and arterial blood pressure did not change from HTD28 onwards, we may hypothesize that atrial pressure, although not directly measured, was chronically reduced (perhaps via reduced blood volume and hence preload) in both groups of subjects. A previous echocardiographic study during bed rest confirmed that the left ventricular end-diastolic volume (a surrogate of cardiac preload) progressively decreases throughout 60 days of bed rest (Westby et al., 2016). Hence, it appears that the main reason for the unchanged HR in the trained subjects could be a training-induced enhancement of vagal tone. SV progressively decreased in both groups during HDT, reaching a minimum at the end of bed rest (i.e., about 90% of the baseline value). The SV reduction is in line with the literature on HDT, which reports a decrease in plasma and blood volume by 10 to 30% within the first 24 to 72 h of confinement (Convertino, 2007). A decrease in SV suggests a dehydration condition, which was probably due to different reasons. One factor was the increased renal sodium excretion and thus reduced water retention (Convertino, 2007). Another factor could be related to tissue compression in lying position that dehydrated areas of weight bearing because of greater interstitial flow into the microcirculation (Hargens and Vico, 2016). The reduced daily physical activity might also have been a cause of dehydration (Convertino, 2007).

The training not only failed in counteracting the decrease in SV, but it might have even accelerated it. On the 28th day of bed rest, SV was 92% of the baseline value in both groups, but on the 2nd day it was equal to 100% in CTRL participants and decreased to 91% in TRAIN participants. Moreover, on the 2nd day of bed rest SBP decreased significantly only in the TRAIN group, and DBP changes tended to be lower in the TRAIN when compared to the CTRL group (Figures 2E and 2F). This contrast suggests that exercise training might quickly influence the hemodynamic balance, inducing post-exercise hypotension likely due to an early blood volume reduction. We speculate that training might accelerate the loss of plasma volume, as demonstrated by recent works showing that lower limb exercise (cycling) produced a different adaptation of the autonomic sympathetic tone in supine vs. upright position (Ried-Larsen et al., 2017). Supine position activated the stretch receptors of heart, veins, and pulmonary circulation, increasing central blood volume, and blunting the metaboreflex activation. Such a response reduced heart contractility, limiting SV, and reducing blood pressure via a non-renal mechanism. At the same time, this reaction could be responsible for a reduction in plasma renin activity and therefore for an early increase in water loss (Ried-Larsen et al., 2017). Thus, a potential additive effect on the cardiovascular system of acute blood volume changes and sympathetic response to exercise might occur in the first days of training and HDT. We can suggest that, in order to prevent such additive effect, the exercise-based countermeasures should start at full load a few days after exposure to microgravity or its analog. The only other time point at which we observed a significant difference in blood pressure between groups was 10 days after the end of HDT (R+10), when CTRL participants showed a decrease in supine SBP compared to baseline of 17 mmHg (see Table 2), a marked phenomenon of hypotension absent in the TRAIN group. As will be discussed later, this suggested that prolonged bed rest had long-term effects on blood pressure control mechanisms, potentially leading to orthostatic hypotension at the restoration of normal gravity conditions. The implemented training protocol showed positive effects on these cardiovascular modifications. With respect to the cardiac autonomic modulations of HR, previous studies documented a decrease in HRV total power and vagal indices in early and chronic HDT (Fortrat et al., 1998; Sigaudo et al., 1998; Pavy-Le Traon et al., 2007), as well as contrasting findings on the sympathetic cardiovascular control (Hughson et al., 1994; Sigaudo et al., 1998; Fortrat et al., 2001; Ferretti et al., 2009). In our study, the HF power decreased significantly in the CTRL group on the 28th and 56th day of bed rest (Figure 3). The breathing rate was stable before, during, and after HDT, always falling within the HF band (Table 2). In our experimental set-up, the HF power thus correctly represented the respiratory component of the parasympathetic modulations of HR. Therefore, our data indicated that HDT induced a substantial reduction in vagal modulations of HR in the respiratory band. The LF/HF powers ratio is an index of cardiac sympathovagal balance: in the CTRL group, it tended to increase on the 28th and 56th day (+9 and +13% after log transformation, Figure 3F). Additionally, DFA1 quantifies changes in the sympathovagal balance, but unlike the LF/HF powers ratio, it considers fractal components of the HR dynamics not related to the amplitude of the oscillations. Concurrently with the HF power reductions (Figure 3A), DFA1 also tended to increase in the CTRL group on the 28th and 56th day of bed rest and remained higher than baseline even 10 days (i.e., R+10) after the end of bed rest (Figure 3D). The LF power reflects both the vagal and sympathetic cardiac modulations; in CTRL participants it decreased in a similar fashion as the HF power, suggesting the predominance of the vagal withdrawal compared to possible sympathetic activation (Figure 3B). The VLF power reflects the cardiac modulations of different humoral and thermoregulatory mechanisms superimposed on the autonomic cardiac control; the time course of the VLF power during bed rest was remarkably similar in the two groups, suggesting that the effects of training on the changes in HR variability during bed rest mainly regards the fast-vagal modulations of HR (Figure 3C). Unlike DFA1 or the LF/HF powers ratio, the power of DBP oscillations in the LF band is a measure of sympathetic vascular control not influenced by parasympathetic modulations. This index did not show any significant effect of bed rest (Figure 3E). Therefore, the analysis of changes occurring during bed rest in the cardiovascular autonomic indices suggested a reduction of vagal heart rate modulations and an increase in the sympathovagal balance without evidence of an altered sympathetic tone in the CTRL group. This effect was detectable up to 10 days after the end of bed rest. The trends were different in the TRAIN group without apparent alterations during the HDT period with regard to any autonomic index. Therefore, our results suggested that during HDT the reactive jumps training reduced the cardiac autonomic deconditioning.

### Hemodynamic and autonomic response to the postural test

The postural test allowed for the evaluation of the effects of the 60-day HDT on the cardiovascular system as it operates around different working points. In the supine position, the upper part of the body contains a larger volume of fluids than in sitting position. The fluid shift from sitting to supine posture is expected to increase the volume of the large vessels and the filling of the heart chambers, stimulating volume receptors which induce an autonomic response. The descriptive statistics of hemodynamic variables and autonomic indices in Table 2 showed higher SV and vagal index, and lower HR, sympatho/vagal balance indices, and TPR in supine compared to a sitting position. Our results indicate that in the recovery phase 10 days after bed rest, the cardiovascular deconditioning affects some variables more in one position than in the other. For instance, this is the case with HR (Figure 4A) because the HR *delta score* of the CTRL group significantly differed from zero in only the supine position. Such a finding points out that after HDT without countermeasures, the cardiovascular deconditioning affects HR more in supine than in sitting position. HR *delta scores* of TRAIN participants were indeed closer to zero in both positions and lower than those of the CTRL group in the supine position (Figure 4A). This finding confirms the efficacy of the administered training protocol as a countermeasure for cardiovascular deconditioning. In contrast to HR, SV showed the same significant reduction in the recovery phase, between −5 and −7 mL, independent of the posture in both groups, suggesting that the training protocol did not affect the loss of body fluids caused by HDT. Interestingly, the postural test was able to detect a significant reduction in TPR in the CTRL group (Figure 4D), but in the supine position only. The difference between the two postures was also highly significant in the CTRL group. It is possible that the fall in supine TPR induced by the 60-day HDT was responsible for the significant reduction in supine SBP observed at R+10 only in the CTRL group (Figure 2E)—a hypotensive effect also evidenced by the negative *delta score* of supine SBP (Figure 4E). The significant differences between supine and sitting *delta scores* of TPR, SBP, and DBP in CTRL participants further highlighted that long-term effects of head-down-tilt bed rest depended on posture. In this regard, the crucial point is that the sledge jump training protocol showed positive effects. No significant *delta scores* were observed in the TRAIN group for TPR and BP in the supine position, indicating the ability of the administered training protocol to accelerate recovery after HDT bed rest. Interestingly, in the TRAIN group the significantly positive *delta score* of TPR in sitting position (Figure 4D) indicates that exercise training might even have improved the capability to increase the total peripheral resistance in sitting position. An improved endothelial function induced by training (Ashor et al., 2015) could mediate such an effect. The autonomic indices (Figure 5) also shows the effects of HDT related to the posture. When sitting in the recovery phase, the CTRL group had lower sympathovagal activation and vagal withdrawal (Table 2). This is demonstrated statistically by significant negative *delta scores* of the LF/HF powers ratio and DFA1 and by a significant positive *delta score* of the HF power in sitting position (Figure 5). This phenomenon, suggests an impaired autonomic response to a postural shift after prolonged bed rest, a possible marker of orthostatic intolerance. By contrast, the TRAIN group had unchanged (i.e., null) *delta scores* for the indices of sympathovagal balance in both supine and sitting positions, whereas the index of vagal modulations of HR (HF power, Figure 5A) increased after bed rest in a similar manner for both postures. These findings therefore suggest that the proposed training protocol allowed for a faster recovery of the physiological autonomic responses to posture changes.

## Conclusion

Considering that a supine position on Earth mimics acute cardiovascular effects of weightlessness, which induces a robust vagal activation immediately after the fluid shift to the upper body, we described the time course of long-term changes by comparing HDT to supine baseline recordings. Our data revealed different dynamics of cardiovascular adaptations. The acute autonomic changes induced by the supine position persisted throughout short-term HDT exposure and were strongly attenuated during mid-term and long-term HDT exposure, whereas SV adaptations showed an enduring trend. The administered training protocol appeared to mitigate some of the autonomic cardiovascular adaptations occurring during mid- or long-term HDT exposure and to accelerate their recovery after bed rest. The hypotensive phenomenon observed in the TRAIN group only during short-term HDT exposure, however, suggests that administering this exercise with initially light but progressively heavier loads during the first days of bed rest is an effective countermeasure.

## Author contributions

MM, PC and AS wrote the manuscript and processed the data. AS designed and directed the project. KB helped supervise the project and performed data collections with support from AW and SM. PC and MM preprocessed data for statistical analyses. AS performed statistical analyses and prepared the figures and tables. PC, MM, GM, and AS drafted the manuscript. H-CG, LR, MS, and OO provided critical feedback and contributed to the interpretation of the results. All authors discussed the results and contributed to the final manuscript.

### Conflict of interest statement

The authors declare that the research was conducted in the absence of any commercial or financial relationships that could be construed as a potential conflict of interest.
